# The Use of Self-Help Strategies in Obesity Treatment. A Narrative Review Focused on Hypnosis and Mindfulness

**DOI:** 10.1007/s13679-021-00443-z

**Published:** 2021-05-29

**Authors:** Marianna Pellegrini, Sara Carletto, Elena Scumaci, Valentina Ponzo, Luca Ostacoli, Simona Bo

**Affiliations:** 1 Department of Medical Sciences, University of Torino, c.so AM Dogliotti 14, 10126 Torino, Italy; 2 Department of Neuroscience “Rita Levi Montalcini”, University of Torino, Torino, Italy; 3 Department of Clinical and Biological Sciences, University of Torino, Torino, Italy

**Keywords:** Hypnosis, Mindfulness, Obesity, Self-conditioning, Self-help

## Abstract

**Purpose of Review:**

The aim of this narrative review was to summarize the evidence evaluating the possibilities and limitations of self-hypnosis and mindfulness strategies in the treatment of obesity.

**Recent Findings:**

Psychological factors, such as mood disorders and stress, can affect eating behaviors and deeply influence weight gain. Psychological approaches to weight management could increase the motivation and self-control of the patients with obesity, limiting their impulsiveness and inappropriate use of food. The cognitive-behavioral therapy (CBT) represents the cornerstone of obesity treatment, but complementary and self-directed psychological interventions, such as hypnosis and mindfulness, could represent additional strategies to increase the effectiveness of weight loss programs, by improving dysfunctional eating behaviors, self-motivation, and stimulus control.

**Summary:**

Both hypnosis and mindfulness provide a promising therapeutic option by improving weight loss, food awareness, self-acceptance of body image, and limiting food cravings and emotional eating. Greater effectiveness occurs when hypnosis and mindfulness are associated with other psychological therapies in addition to diet and physical activity. Additional research is needed to determine whether these strategies are effective in the long term and whether they can be routinely introduced into the clinical practice.

## Introduction

Obesity is a public health burden [1]. Excess weight is associated with an increased risk for cardiometabolic diseases, cancer, and mortality [2] as well as a range of negative biopsychosocial outcomes and psychiatric symptoms, such as depression and anxiety [3]. The physiopathology of obesity is complex, involving the deregulation of appetite and energy metabolism, genetic, metabolic, biochemical, cultural, and psychosocial factors [4].

Unhealthy diets and poor exercise are considered the main environmental causes of excess weight; psychological factors can indeed heavily influence weight gain [5]. Stress and mood disorders have been linked to increased search for high-density foods, decreased exercise, and altered eating behaviors [5]. “Emotional eating” is the inclination to eat in response to negative emotions, and “external eating” is the tendency to eat in response to external food cues; these behaviors are associated with unhealthy food choices and weight gain [6]. Furthermore, higher rates of depression, low self-esteem, anxiety, eating disorders (binge eating disorder, night eating syndrome, etc.), and impaired health-related quality of life are reported in individuals with obesity [7]. The reward circuits seem to be altered, with a preference for immediate rewards (e.g., high-fat, high-carbohydrate, salty foods) over long-term benefits (e.g., weight management) [8]. Uncontrolled daily stress may alter brain reward/motivation pathways involved in seeking hyperpalatable foods [9]. Indeed, inappropriate eating behaviors may be the result of a poor inhibitory control and hedonic homeostatic dysregulation, which may predispose to overeating palatable food in the absence of hunger [8], up to a condition of food addiction with greater emotion dysregulation, impulsivity, and food cravings [10]. The conditioning model of food cravings states that cravings can develop from pairing consumption of certain foods with external (e.g., watching television) or internal (e.g., feeling sad) stimuli [11].

The management of obesity usually includes lifestyle intervention only as the first approach, even if, in specific cases, based on the severity of the clinical condition, pharmacotherapy may be necessary early for the patient care. The attrition from weight management programs, however, affects most patients [12], and obesity treatment achieves poor results, with a high rate of relapse and weight recovery [2]. The search for approaches addressing the associated psychological problems and potentially increasing the motivation and self-control of the patients with obesity, limiting their impulsiveness and inappropriate use of food, are therefore important. Psychological interventions, particularly behavioral and cognitive-behavioral strategies, have been reported to be beneficial on weight loss in adults with overweight and obesity, especially when combined with dietary and exercise strategies [13]. The cognitive-behavioral therapy (CBT) has proven to be effective in determining weight loss by increasing healthy eating and exercise, improving psychologically related eating behaviors (i.e., cognitive restraint and emotional eating) [14] as well as cognitive factors, such as self-motivation, self-monitoring, and stimulus control [15, 16]. Self-regulation (or self-control) can be defined as the suppression of a behavioral impulse toward a “lower-level” goal in the interest of pursuing a “higher-level” goal [17]. Thus, dietary, and physical activity adherence demands self-regulation, which depends on the ability to maintain a continued awareness of behavior. The lack of this awareness and its consequences result in “mindless” eating and activities [16].

A great interest is related to self-directed psychological interventions that do not require the constant presence of a health professional (“self-help”) [18], while employing the use of manuals, commercial products, technology, supportive peers, or occasional professional assistance [19]. These strategies aim to train patients in skills that enhance self-control such as goal setting, regulation, self-monitoring and evaluation, problem solving skills, and coping strategies for high-risk situations [17]. Several therapeutic approaches alone or in combination include psychodynamic, behavioral, cognitive-behavioral, mindfulness, and hypnotic therapy. The purpose of this narrative review is to focus on the application of hypnotherapy and mindfulness as self-help approaches in the treatment of obesity, replacing or supporting CBT.

## Methods

The following databases were queried: PubMed (National Library of Medicine), Psychological Information Database Medical, Psychiatry, Mental Health Disorders (PsycInfo), Cochrane Library. The search strategy was performed using the following keywords: obesity OR overweight OR weight loss AND self-conditioning, self-control, self-help, self-regulation, strategies, hypnosis, hypnotherapy, hypno-behavioral therapy, mindfulness, cognitive behavioral therapy, psychological treatment, behavioral education. The filters “humans” and “adults” were used. Hand searching the references of the identified studies and reviews was carried out too.

## Hypnosis

### History and Definitions

Hypnosis has been considered as the oldest psychotherapy, being practiced by the ancient Egyptians since the fifteenth century BC. The rediscovery of hypnosis in 1700s is due to the German doctor F.A. Mesmer who noted beneficial effects on the patient discomfort by entering into empathic resonance with him [20]. In 1841 the English doctor J. Braid introduced the term “hypnotism,” based on the physiology of the brain [21, 22]. Due to the theories of J.M. Charcot and his most famous student, S. Freud, who considered the hypnosis as a pathological phenomenon, an artificial hysterical neurosis with a limited therapeutic value, the hypnotic method was abandoned [20, 23, 24]. During the world wars, hypnosis experienced a renewed interest, as it was applied to treat the war traumatic neuroses. More recently, the psychiatrist M. Erickson (1901–1980), by elaborating the concept of the unconscious, applied the clinical hypnosis in the re-elaboration of negative or traumatic events associated with adverse symptoms or diseases [20–25].

Although hypnosis acquired scientific dignity among scientists and clinicians, no agreement on its definition has been achieved [20, 25]. The British Psychological Society defines “hypnosis” as a waking state in which the individual attention is focused away from his/her surroundings and absorbed by inner experiences such as feelings, cognitions, and imagery, which can be influenced by the interaction with a “hypnotist” [26]. According to the American Psychological Association, hypnosis is a procedure during which a hypnotist guides the subject to respond to suggestions for changes in subjective experience, alterations in perceptions, sensations, emotions, thoughts, or behaviors [27, 28]. The neo-Ericksonians defined hypnosis as a strategy that explores the deeper causes of the disorders rather than aiming at the remission of the symptoms; indeed, during the hypnotic trance, the unconscious offers the possibility of solutions to problems or conflicts [20, 25–29].

### The Rationale for Using Hypnosis in the Treatment of Obesity

**Fig. 1:**
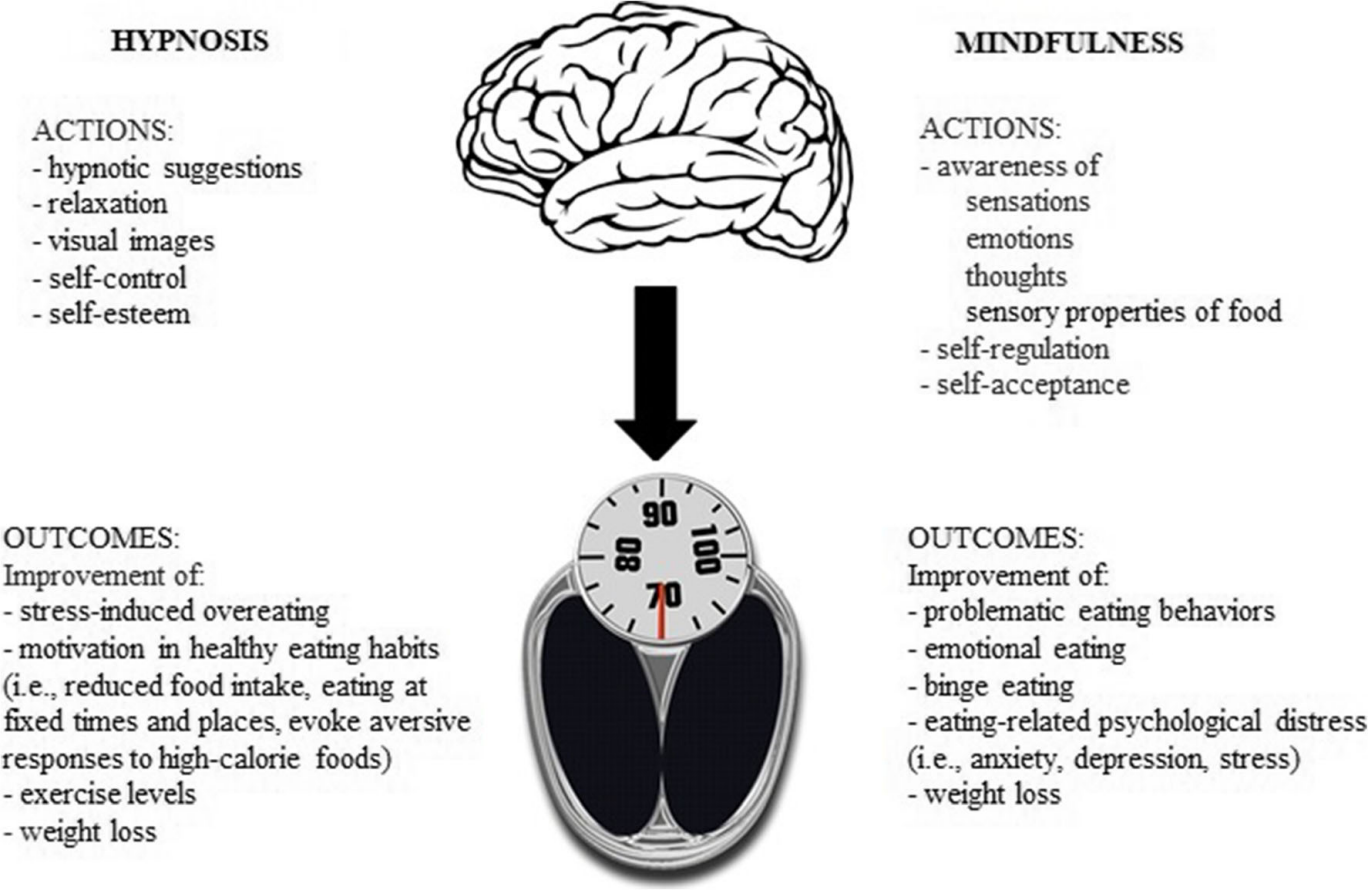
Hypnosis and mindfulness as strategies for stress management in obesity treatment

Hypnosis has been successfully used as an anti-stress and relaxation strategy to treat many chronic conditions exacerbated by negative emotions and social factors (e.g., quitting smoking [30]), chronic digestive diseases [31], cancer-related symptoms in palliative care setting [32], acute and chronic pain [33]. Individuals can practice hypnosis on their own (self-hypnosis); hypnotherapy (either alone or in addition to other psychotherapies, such as cognitive-behavioral techniques) has been involved in the obesity multidimensional approach to increase self-control, to improve exercise levels, to boost self-esteem, and to strengthen motivation in changing eating habits [34]. Stress-induced overeating and reward from comfort food can be considered an attempt at self-medication to relieve the negative emotions and depressive state associated with chronic psychological stress [35]. Hypnosis as a strategy for stress management could be an important component in the approach toward stress-induced overeating and impaired reward mechanisms characterizing many patients with obesity (Fig. 1).

### Studies on Hypnosis and Weight Management

The application of hypnosis for weight reduction has been reported in the literature since the 1950s [36]. Hypnotized state was initially induced by experienced therapists; indeed, self-hypnosis and the autonomous use of supplemental materials (i.e., home audio tapes) have been encouraged from the earliest years of hypnotherapy to reinforce the therapist suggestions and provide additional support after the formal treatment completion [37]. With hypnotic suggestions (i.e., verbal, and non-verbal communications), people can be taught to reduce food intake, eat at fixed times and places, restrict the purchase of food supplies, and evoke aversive responses, such as sickness and disgust, when eating high-calorie foods [34]. Clinical trials comparing cognitive-behavioral therapy alone versus cognitive behavioral therapy plus hypnosis for various conditions, including obesity, were first reviewed in 1990s in two meta-analyses [38, 39]. Kirsch [38] analyzed 6 weight loss trials and reported an improvement in weight loss with hypnosis (weighted mean effect size=1.96) and no weight regain after hypnosis even at 2-year follow-up. However, this meta-analysis [38] displayed several methodological limitations (lack of data availability for some studies, high risk of bias of the included research, short follow-up for most of the included studies, high drop-out rates). Allison [39] re-analyzed the same 6 weight loss trials and found that hypnosis, as an adjunct to CBT, produced only a small effect on average (weighted mean effect size=0.28) [39]. Kirsch [40], then, re-conducted the metanalysis and reported an effect size of 0.98, which was different from previous results, but still indicative of a benefit from the combination of hypnosis with CBT. The correlation between the efficacy of hypnosis in weight loss program and the degree of hypnotizability is highly controversial, since not all studies found such a relationship. The mechanism by which hypnotherapy might work is the processing of problems related to nutrition through the detection and integration of neglected resources, relaxation, hypnotic suggestions, and visual images [41]. In 1986, a RCT assessed in 60 overweight women the effectiveness of 1-month hypnosis on weight loss, either alone (Hy; *n*=17 patients) or plus audiotapes (Hy-T; *n*=17 patients), compared to a wait-listed control group (*n*=20 patients) [42]. When compared to controls, both the Hy and Hy-T interventions significantly reduced body weight after 1 month (−3.62 kg Hy; −2.96 kg Hy-T; +0.68 kg controls; *p*<0.01) and 6 months (−7.76 kg Hy; −8.00 kg Hy-T; −0.22 kg controls; *p*<0.01) [42]. In 1998, 60 individuals with obesity and sleep obstructive apnea (80% males) were randomized to receive either hypnotherapy for the reduction of stress or hypnotherapy for reducing energy intake or dietary advice alone for 18 months [43]. All the three groups lost 2–3% of their initial body weight at 3 months, while, at 18 months, only hypnotherapy for stress reduction determined a significant (*p*<0.02), but small weight loss (3.8 kg) compared to baseline. Noteworthy the dropout rate was 25% [43], thus requiring caution in the interpretation of these results. In 2014, two types of hypnotherapies were compared in 60 females with obesity by a parallel RCT: hypno-behavioral therapy (HypBe) (a combination of hypnotherapy—i.e., hypnotic trances—plus behavioral therapy—i.e., behavioral exercises, role-playing, and homework) and hypno-energetic (HypEn) therapy (a therapy enhancing the hypno-behavioral strategies with acupressure, that is, the manual stimulation of acupuncture in the related points) [44]. Both treatments consisted of 12 sessions lasting 120 min over an 8.5-month period, and participants were assessed at the beginning and the end of the treatment, as well as after 6-month follow-up. Regardless of the hypnotherapy received, initial weight and BMI were significantly reduced from the beginning to the end of treatment (−2.4 kg, *p*<0.01 and −0.8 kg/m^2^, *p*<0.01 within the HypBe group; −3.1 kg, *p*<0.01 and −1.1 kg/m^2^, *p*<0.01 within the HypEn group), but significant weight and BMI reductions from the end of treatment to the follow-up (−2.2 kg, *p*<0.01 and −0.8 kg/m^2^, *p*<0.01) and significant improvements in eating behaviors, and several aspects of body concept, such as physical efficiency, self-acceptance of the body/physical appearance/sexuality, were observed in the HypEn group only [44]. A systematic review of 5 meta-analyses of RCTs demonstrated the efficacy of medical hypnosis in the reduction of pain and emotional stress during medical interventions (34 RCTs, 2597 patients) [45]. Indeed, the application of hypnotherapy aims to emotionally restructure stressful events and sensations and cognitive–affective patterns (through minimization, reinforcement, new conditioning), and to improve problem management by giving the patient access to his own resources, thus facilitating changes in behaviors [45]. Milling performed two meta-analyses comparing hypnosis with a control condition including standard care, attention control, or no-treatment (14 trials) and CBT alone with CBT augmented by hypnosis (11 trials) over a relatively short span of time (average length of the hypnosis interventions in the two samples of trials ≈ 6.5 weeks) [46••]. Hypnosis demonstrated to be more effective in producing weight loss when compared to the control condition (effect sizes=1.58 lb, *p*≤0.001 at the end of the active treatment in 14 trials, and 0.88 lb, *p* ≤0.001 in 6 trials with longer follow-up) [46••]. Similarly, hypnosis plus CBT compared to CBT alone induced an increased weight loss (effect sizes=0.25 lb, *p*≤0.05 in 11 trials at the end of the intervention and 0.80 lb, *p*≤0.001 in 12 trials with longer follow-up of ≈ 12 weeks) [46••]. Recently, a RCT evaluated the effectiveness of self-hypnosis added to standard care in determining weight loss in patients with severe obesity [47]. A rapid-induction phase was used to allow the patient to go into hypnosis in a few minutes, then participants were trained to enter into hypnosis in complete autonomy daily, before each meal [47]. In the self-hypnosis arm, a significant improvement in quality of life, satiety, and inflammation occurred with respect to controls with standard care, without a significant difference in weight loss (−6.5-kg intervention group, *n*=44 patients; −5.6-kg control group, *n*=42 patients; *p*=0.79). Indeed, within the intervention group, habitual hypnosis users showed a greater weight loss than those who practiced self-hypnosis less frequently (−9.6 kg, ≥ once per day; −7.5 kg <once per day; +0.2 rarely or none; *p*=0.001) [48]. The same research group reported an acute effect on the brain peptides involved in the hunger/satiety regulation after a hypnosis-induced hallucinated meal in highly hypnotizable individuals, thus suggesting the potential role of hypnosis on central appetite modulation [49•].

In conclusion, 11 randomized trials about the effect of hypnosis as an adjunctive strategy to lose weight were published; out of them, 9 [42–44, 48, 50–54] reported overall beneficial effects, even if mild or moderate and more evident after a longer follow-up [42, 44, 50, 51, 53], while only 2 [55, 56] failed to find any benefits.

A few ongoing trials studying the use of hypnosis in the treatment of obesity [57, 58] are reported in Table 1.

**Table 1:** Ongoing trials on hypnosis and obesity [57]

Study title	Country	Intervention	Status	Additional information
Hypnosis, Self-hypnosis, and Weight Loss in Obese Patients	France	- Dietetic counseling - Hypnosis and self-hypnosis	Completed	No published data retrieved
Efficacy of Self-hypnosis for Weight Loss in Type 2 Diabetics	USA	- Self-hypnosis - CDE training - No special treatment (control)	Completed	After 1 year, weight loss was −2.7 kg (self-hypnosis; *n=*36 patients), −1.8 kg (CDE; *n*=38 patients), and −0.45 (controls; *n=*102 patients), *p*=0.001 [58]
Changing Eating Behaviours of Healthy Adults Through Hypnosis	Romania	- Hypnosis with amnesia suggestions - Hypnosis with cognitive rehearsal suggestions - Hypnosis with memory substitution suggestions - Hypnosis with induction only	Completed	No published data retrieved
The Impact of the Hypnosis on the Loss of Weight at Patients in Failure of Bariatric Surgery	France	- Hypnosis - Standard care	Recruiting	-
Hypnosis and States of Change to Promote Weight Loss	Lebanon	- Listening to an audiotape	Recruiting	-
Changing Eating Behaviour Using Cognitive Training	Romania	- Hypnosis - Food inhibition training - Control	Not yet recruiting	-

*CDE* Certified Diabetes Educator

### Limitations of Hypnosis as Self-Help Strategies to Lose Weight

Although hypnosis has been reported as a successful approach to promote weight loss in addition to lifestyle and/or psychological interventions, the overall number of related studies was low, and most trials were very old, i.e., published in the 1980s [42, 50–53, 55, 56]. Furthermore, the methodological quality was limited; most studies had an observational design, a low number of enrolled patients, short duration of the intervention, and only a few reported follow-up data. Patient recruitment might be a challenge in hypnosis-based interventions, the individual hypnotizability is still a highly debated question [41], and the available papers reported an increased female participation, with high rates of drop-out or discontinuation of the intervention [43, 44, 48]. Due to the small sample sizes, some studies might lack the statistical power to detect differences between the interventions [54], and divergent effect size values were reported in meta-analyses [38–40, 44, 46]. Therefore, at present, the evidence toward the efficacy of hypnosis as a strategy for losing weight is scarce.

### New Technologies

Due to the great interest in self-managed strategies that could help individuals to lose weight, an increasing number of internet websites, videos, and smartphone applications dedicated to hypnosis are available. A review of 407 hypnosis applications for smartphones and tablets reported that the most frequently proposed goal was weight loss (22.6%), by delivering hypnosis via audio track, visual means (i.e., reading the text of a hypnosis script), or both [59]. However, only a few applications mentioned the hypnotist being a “doctor,” and none reported being evidence-based (empirically tested); therefore, concern about their safety and efficacy was reported [59]. Further rigorous studies to support the effectiveness of hypnosis applications are needed in order to develop scientifically validated tools for consumers.

## Mindfulness

### History and Definitions

Mindfulness practice has a very long history, dating back to over 2500 years ago. It was part of different religious and secular traditions, such as Hinduism, Buddhism, and yoga [60], but became known in the West in the 1970s thanks to Kabat-Zinn, who abstracted the concept from its original religious context [61]. Mindfulness could be defined as a non-judgmental awareness and acceptance of one’s moment-to-moment experience [62]. As described by Bishop [63], mindfulness consists of two main components: the self-regulation of attention and a particular orientation toward the experience. Self-regulation of attention concerns the non-judgmental observation and awareness of physical sensations, affective states, and thoughts as they arise. Orientation to experience refers to the attitude of acceptance and curiosity toward one’s experience. Mindfulness has been described as a set of skills that can be learned through practices like meditation and therapeutic interventions, the Mindfulness-Based Interventions (MBIs). MBIs are usually conducted in groups, and participants are guided by instructors who have received specific training and have a personal meditation background, but the fundamental and distinctive element remains the experiential learning that each participant acquires during the course by means of daily training.

Mindfulness-Based Stress Reduction Program (MBSR) training is the most common form of MBIs and consists of eight 2.5-h weekly sessions and one 7-h day of silence [62, 64]. This training aims at improving the capacity of attention and non-judgmental awareness and could teach people to break with the maladaptive patterns of thinking and behavior. These maladaptive strategies are believed to contribute to the onset and maintenance of many emotional disorders [63]. Alongside with MBSR, Segal [65] proposed a Mindfulness-Based Cognitive Therapy (MBCT) program, that is an approach which brings together elements from MBSR and from Cognitive Therapy, aiming to prevent depressive relapse and depressive symptoms. Mindfulness-Based Eating Awareness Training (MB-EAT) is a specific mindful eating training program integrating MBSR and CBT components [66, 67]. It was originally designed for binge-eating disorder, but it was also implemented in non-clinical populations to promote weight loss. MB-EAT is aimed at improving eating behavior and more generally at developing a healthier and more balanced attitude toward food. Attention is also paid to stress related to eating and dysfunctional modes of eating behavior (e.g., emotional, and external eating).

### The Rationale for Using Mindfulness-Based Interventions in the Treatment of Obesity

Mindfulness practice has been repeatedly reported to decrease global psychological distress and improve overall mental health [68, 69]. MBIs have also shown their effectiveness in decreasing anxiety, as well as improving depressive symptoms [70], by ameliorating self-regulation [71].

Different dysfunctional eating behaviors, such as binge eating, emotional eating, external eating, and eating in response to food cravings, have been linked also to weight regain after successful weight loss [72]. Furthermore, the distracted and unaware eating impairs the memory of the meal and increases further food intake, suggesting that the attentive and mindful experience of a meal is necessary for adequate satiation mechanisms and proper inhibitory controls [35]. The different theoretical models for explaining problematic eating behaviors suggest the association between maladaptive responses to internal and external stimuli and the dysregulation of eating behaviors [72, 73]. Several studies have also indicated stress and negative emotions among the principal determinants of unhealthy eating behavior [74–76]. As mindfulness and mindful eating promote self-regulation by better management of negative emotional states and stress, and promote awareness of the sensations associated with eating, these trainings can be used as strategies to help people to diminish the reactivity to dysfunctional food cues (e.g., advertising, boredom, anger, anxiety) [77] and to favor weight control (Fig. 1).

### Studies on Mindfulness-Based Interventions and Weight Management

The first integrative review on the role of MBIs in the treatment of obesity was published in 2013 [78]. While denoting the paucity of studies conducted up to that time, this review provided a general overview of the use of MBIs either as stand-alone treatment or as complementary to other traditional approaches in the treatment of obesity and eating disorders. O’Reilly [72] conducted a literature review to investigate the effects of MBIs to treat obesity-related eating behaviors (e.g., binge eating, emotional eating, and external eating) on 21 primary studies, showing that in 86% of the included studies there was an improvement in targeted eating behaviors. The first systematic review [79] included 14 interventional studies and found a reduction in binge and emotional eating after mindfulness meditation training. Another systematic review [80] reported that 13 of the 19 included studies showed a beneficial effect of MBIs on weight loss, although the specific degree to which increased mindfulness was a mechanism leading to weight loss was not identified. In fact, mixed results were found regarding the association between mindfulness change (i.e., after MBIs) and weight loss. Further research to investigate the specific mechanisms involved in the relationship between mindfulness and weight loss was recommended. Tapper [81] attributed the beneficial effects of MBIs on weight loss and impaired eating behaviors to both the present moment awareness of the sensory properties of food that can reduce further food intake, and the decentering strategies that may help individuals resist desired foods. The first review that considered the change in mindfulness as a primary outcome and mindful eating as a measured variable was performed by Dunn in 2018 [82]; the authors strongly supported the use of mindfulness in weight management programs, also suggesting a potential benefit for the treatment of obesity. A very recent systematic review [83], including 9 RCTs, showed that in most of the included studies, there was a positive effect of MBIs on reducing emotional eating, binge eating, and weight and shape concern. The mechanisms of action highlighted were the increase in awareness of internal experiences and automatic patterns and the improvement in self-acceptance and emotional regulation, which led to a reduction of problematic eating behaviors. Moreover, to date six meta-analyses were published evaluating the efficacy of MBIs for weight management, obesity, and/or problematic eating behaviors. Overall, their results showed that MBIs have beneficial effects on both obesity-related eating behaviors [84, 85] and binge eating [86, 87]. In particular, moderate-to-large effect sizes (Hedge’s g ranging from 0.70 to 1.08) were found for obesity-related eating behaviors [84, 85], while large effect sizes have been shown for binge eating (Hedge’s g ranging from −0.90 to −1.08) [86, 87]. The effect on weight loss was found to be moderate in both Carrière [84] and Rogers [85] meta-analyses, with Hedge’s g =0.42 and Hedge’s g =0.47, respectively. The greater effects on weight loss were found in studies that used a combination of informal and formal meditation practice rather than formal meditation practice alone [84]. In another meta-analysis [88], a significant weight loss effect of MBIs was found when compared with non-intervention controls (standardized mean difference: −0.348 kg, 95% CI: −0.591 to −0.105, *p* = 0.005), showing that the MBI effect was similar to common diet programs. Lawlor [89] recently performed a network meta-analysis to evaluate the effect of third-wave cognitive behavior therapies for weight management. Their result pointed out that Acceptance and Commitment Therapy (ACT) intervention was the intervention with the greatest effect on weight loss as compared to standard behavioral programs, with long-lasting effects at 12- and 24-month follow-up. Moderate effects of MBIs were also revealed for the impact on eating-related psychological distress, with a Hedge’s g =0.64 for depression and 0.62 for anxiety [85]. Several other trials on mindfulness in weight management are ongoing [90–92] (Table 2). Recent studies conducted in this area, which were not included in the reviews and meta-analyses above described, are shown in Table 3 [93–107]. Sixteen studies were retrieved, including 9 RCTs and 7 intervention studies without a control group. Overall, these studies showed promising findings supporting the effectiveness of MBIs to improve problematic eating behaviors and weight management.

**Table 2:** Ongoing trials on mindfulness and obesity [90]

Study title	Country	Intervention	Status	Additional information
Effect of Self-Regulation with Mindfulness Training on Body Mass Index and Cardiovascular Risk Markers in Obese Adults	USA	- Dietary counseling - Mindfulness training program	Completed	No published data retrieved
Psycho-sensorial Mindfulness and Top-down Control: Mindfulness Program for Obese Patients in Preparation to Bariatric Surgery	France	- Bariatric surgery with mindfulness program - Bariatric surgery without mindfulness program	Completed	No published data retrieved
Nutritional Video Intervention Using Mindfulness-based Principles	USA	- Healthy cart and stress management videos (2-video group) - Healthy cart video (1 video group)	Completed	At 2-month follow-up, knowledge improved in both intervention groups (*p*<0.001). The 2-video group (*n*=29 women) improved more in self-efficacy and use of a shopping list (both *p*<0.05) and purchased more healthy foods (*p*<0.05) than the 1-video group (*n*=39 women) [91].
Engaging Motivation for the Prevention of Weight Regain	USA	- Mindfulness-based weight loss maintenance - Standard behavioral weight loss maintenance	Completed	No published data retrieved
The Effects of Mindfulness Training on Eating Behaviors and Food Intake	USA	- Mindful eating and living course	Completed	No published data retrieved
Mindful Eating and Living for Obese Women	USA	- Mindful eating and living - Active weight loss control	Completed	No published data retrieved
Craving and Lifestyle Management Through Mindfulness Study	USA	- Craving and lifestyle management through mindfulness	Completed	Outcome data are presented on clinicaltrials.gov [92], but no published data were retrieved.
Mindful Construal Diaries: Can the MCD Increase Mindfulness and Mindful Eating in Bariatric Surgery Patients	United Kingdom	- Mindful construal diary (MCD)	Completed	No published data retrieved
Food Insecurity, Obesity, and Impulsive Food Choice	USA	- Mindful eating - Nutrition DVD	Completed	No published data retrieved
Trauma Exposure, Emotion Regulation and Eating Pathology in Obese Patients	France	Not reported	Completed	No published data retrieved
Efficacy of Mindful Tai Chi on Obese or Overweight Adults: A Randomized Controlled Clinical Trial	USA	- Mindful Tai Chi intervention - Mindfulness meditation - Mall walking - Weekly discussion	Terminated	-
The Role of Values, Acceptance, and Mindfulness Strategies in Long Term Weight Management	Canada	- Acceptance and commitment therapy	Recruiting	-
Project Activate: Mindfulness and Acceptance Based Behavioral Treatment for Weight Loss	USA	- Behavioral treatment - Mindful acceptance - Values - Mindful awareness	Recruiting	-
Mindfulness and Compassion-based Programs on Food Behavior of Patients with Weight Regain After Bariatric Surgery	Brazil	- Mindfulness-based health promotion + treatment as usual - Attachment-based compassion therapy + treatment as usual - Treatment as usual	Recruiting	-
Effect of a Group Intervention Program Based on Acceptance and Mindfulness on the Physical and Emotional Well-being of Overweight and Obese Individuals	Spain	- Standard + acceptance and mindfulness-based group intervention program - Standard	Active, not recruiting	-
The Impact of 8 Weeks of Digital Meditation Application and Healthy Eating Program on Work Stress and Health Outcomes	USA	- Meditation - Healthy eating - Meditation + healthy eating	Active, not recruiting	-
Brief mHealth Self-Compassion Intervention on Internalized Weight Bias	USA	- Self-compassion mindfulness practice	Active, not recruiting	-

*BMI* body mass index

**Table 3:** Recent RCT on mindfulness in weight management programs.

Author, year [ref]	Study design	Participants	Number	Mindfulness strategy	Main results	Specific results on weight loss
Daubenmier, 2020 [93]	RCT	Adults with obesity	194	*n*=100: mindfulness training (meditation, mindful eating, mindful walking) with diet-exercise intervention *n*=94: only diet-exercise intervention	Mindfulness participants showed significantly greater maintenance of challenge-related emotions and cardiovascular reactivity patterns, independently from changes in BMI	No significant group differences between intervention groups were found in 3-month weight loss.
Radin, 2020 [94]	RCT	Adults with obesity	194	*n*=100: mindfulness training (meditation, mindful eating, mindful walking) with diet-exercise intervention *n*=94: only diet-exercise intervention	Participants with higher compulsive eating at baseline randomized to the mindfulness intervention had greater improvements in fasting blood glucose at 18 months	Weight loss at 18 months in both intervention groups was associated with a reduction in stress and compulsive eating at 6 months.
Levin, 2020 [95]	RCT	Adults with overweight and obesity	79	*n*=39: ACT on health online course and coaching calls *n*=40: waiting list	Participants in the ACT condition improved significantly the healthy eating index and the outcomes assessing self-reported eating behaviors, weight, mental health, weight self-stigma, and psychological inflexibility	A greater improvement on self-reported weight was found in participants assigned to ACT condition than the waiting list.
Czepczor-Bernat, 2020 [96]	Intervention study	Adult women with overweight and obesity	184	*n*=184 mindful eating	Mindful eating was a significant moderator for emotional eating, and restrictive eating, but not for uncontrolled eating; mindful eating was a significant moderator for the relationship between negative emotions and emotional eating, restrictive eating, and uncontrolled eating	-
Felske 2020, [97]	Proof-of -concept intervention study	Adults with obesity seeking bariatric surgery	56	*n*=56: MII sessions with cognitive, behavioral, and psychoeducational components	Improvements in addictive-like eating, binge eating, emotional eating, and grazing were observed from pre- to post-MII and at 12-week follow-up	-
Schnepper, 2019 [98]	RCT	Individuals motivated to improve their eating behavior or lose weight	46	*n*=23: mindfulness-based training and prolonged chewing intervention *n*=23: waiting list	Participants in the intervention group significantly reduced BMI, emotional eating, external eating, and food cravings.	The intervention decreased BMI, and this loss was maintained during 4 weeks of follow-up.
Pinto-Gouveia, 2019 [99]	Intervention study	Women with overweight or obesity and binge eating disorder	31	*n*=31: BEfree program, a 12-session group intervention that integrates psychoeducation, mindfulness, compassion, and value-based action	Participants in Befree program decreased in binge eating severity, eating psychopathology, external shame, self-criticism, psychological inflexibility, body image cognitive fusion, and increased self-compassion and engagement with valued actions. These results were maintained at 3- and 6-month follow-up.	A significant decrease in BMI after intervention was observed, even though weight loss was not identified as BEfree’s primary outcome.
Jastreboff, 2018 [100]	Pilot RCT	Low-income parent-child dyads with parent obesity	42 dyads	*n*=20: mindfulness-based parent stress group intervention (parenting mindfully for health) + nutrition and physical activity counseling (PMH+N) *n*=22: control group intervention (C+N)	Compared with the C+N group, participants in the PMH+N group demonstrated a significant reduction of parental emotional eating rating. Only participants in C+N showed a significant increase in child body mass index percentile during treatment.	Findings indicate a greater increase in child BMI percentile for the C+N group vs the PMH+N group at post intervention. No significant differences between groups were found for parent BMI.
Hanson, 2018 [101]	Intervention study	Patients attending a tier 3-based obesity and weight-management service	66	*n*=33: mindfulness-based group intervention (mindfulness-based eating behavior strategies taught in four group sessions) *n*=33: retrospective control group	Participants in the mindfulness-based group intervention significantly improved in self-reported eating behavior (particularly fast-foodism) and in self-esteem and confidence in self-management of body weight.	A significant weight loss (3.06 kg, SD 5.2 kg) over 6 months was observed in the mindfulness group as compared to the control group.
Wnuk, 2018 [102]	Intervention study (feasibility pilot study)	Post-bariatric surgery women	28	*n*=28: mindfulness-based eating awareness training (MB-EAT)	Significant reduction of depression and improvement in emotion regulation were observed. The amount of mindfulness practice between sessions resulted associated with statistically significant improvements in emotional eating in response to anger.	Participants maintained their BMIs from pre- to post-intervention.
Spadaro, 2017 [103]	RCT	Overweight and obese adults	46	*n*=24: standard behavioral weight loss program (SBWP) only *n*=22: SBWP + mindfulness meditation (MM)	Participants in the SBWP+MM group significantly reduced their weight and improved eating behaviors and dietary restraint, as compared to SBWP alone.	Enhanced weight loss by 2.8 kg was observed in the SBWP+MM group as compared to SBWP.
Adler, 2017 [104]	RCT	Adults with a BMI in the range 30–45 kg/m^2^	194	*n*=100: mindfulness-based eating intervention (with meditation practices modeled on MBSR, and mindful eating practices modeled on the Mindfulness-Based Eating Awareness Training program) *n*=94: progressive muscle relaxation (PMR)	No significant differences found in sleep quality between the participants in the mindfulness group and the active control group, despite sleep improving from baseline to 6 and 12 months in the mindfulness group. Within the mindfulness group, the amount of mindfulness practice was associated with improved sleep quality.	Both groups experienced reduction in BMI, but change in BMI from 0 to 6 months was not associated with change in sleep quality in either group.
Palmeira, 2017 [105]	RCT	Women with overweight or obesity	73	*n*=36: Kg-Free intervention based on mindfulness, ACT, and compassion approaches *n*=37: Treatment as Usual (TAU)	Participants in Kg-Free intervention significantly reduced weight-related negative experiences and improved their healthy behaviors, psychological functioning, and QoL, as compared to TAU. No significant differences were found between groups regarding self-compassion.	Kg-Free group revealed a reduction of BMI at post-treatment, albeit with a rather small effect size.
Raja-Khan, 2017 [106]	RCT	Women with overweight or obesity	86	*n*=42: MBSR *n*=44: health education	Participants in the MBSR group, as compared to the control group, showed a significant improvement in mindfulness and a significant reduction of perceived stress and fasting glucose. No significant changes in blood pressure, weight, or insulin resistance were observed.	No significant change in weight in the MBSR group was observed.
Levoy, 2017 [107]	Intervention study (exploratory study)	Adult individuals	317	*n*=317 MBSR program	Participants in MBSR showed a significant reduction of emotional eating scores. Changes in mindfulness were correlated with changes in emotional eating.	There were no significant changes in BMI, and baseline BMI predicted weight changes post-MBSR.

*ACT* acceptance and commitment therapy, *BMI* body mass index, *MII* mindfulness-informed, *MBSR* mindfulness-based stress reduction, *n* number, *RCT* randomized controlled trial, intervention, *TAU* treatment as usual

### Limitations of Mindfulness as Self-Help Strategies to Lose Weight

The main limitations of the research conducted so far are represented by the high heterogeneity of included cohorts and the different MBIs programs among the studies. Combining clinical and non-clinical populations in the same quantitative synthesis could be problematic as MBIs may exert different effects in these two groups [84]. It is very important to consider that different types of mindfulness training could be grouped under the “MBIs” label [84]. These include combined mindfulness and cognitive behavioral therapies, MBSR, mindful eating programs such as MB-EAT, third-wave cognitive behavior therapies (e.g., ACT), dialectical behavior therapy-DBT, MBCT, compassion-focused therapy-CFT, and other different combinations of mindfulness exercises. In addition, these interventions may vary in terms of therapeutic components, length, and time of practice requested to participants. Another main limitation is the use of small sample sizes and limited follow-up assessments. In order to strengthen the evidence and evaluate possible long-lasting effects, more research with larger sample sizes and with longer follow-ups is needed. More RCTs are also needed to compare MBIs with other active interventions (e.g., conventional diet programs, standard behavioral treatment, dietary counseling) in order to assess the specific effects of mindfulness on weight management and dysfunction eating behaviors. In this regard, few studies included a valid measurement of mindfulness skills [84, 87], which is fundamental to evaluate both the extent to which increased mindfulness is an active component of treatment and the underlying mechanisms by which MBIs may improve weight management and associated psychological factors [85–87].

### New Technologies

Empirical evidence suggests that MBIs can be effectively delivered online, with significant effects in reducing stress, depression, anxiety, and in improving quality of life in both non-clinical and clinical samples [108, 109]. Lyzwinski performed two interesting reviews evaluating the quality and the effects of electronic MBIs for weight-related behaviors [76, 110]. They reviewed all the commercial mindful eating applications available on Apple iTunes until 2018 to evaluate their quality and the adherence of their contents to the fundamental tenets of mindful eating [110]. Most of the applications revealed a poor-quality score according to the Mobile App Rating Scale (MARS), and few were found to include the essential aspects of mindful eating, although they claimed to do so. Moreover, a systematic review assessed the effects of electronic MBIs for weight and weight-related behaviors [110]. Among the 21 included studies, MBSR protocol was used in 19 studies and mindful/intuitive eating interventions in the other two. Most of the electronic interventions were aimed at stress management, and only a few targeted at weight control. The results showed beneficial results for stress reduction. Based on this very limited number of studies, however, it was not possible to evaluate the impact of these online mindfulness trainings on weight management. Further studies that directly target weight-related behaviors and evaluate other types of mindfulness-based approaches are needed to establish the efficacy of online MBIs in this area.

## Similarities and Differences Between Hypnosis and Mindfulness

Hypnosis and mindfulness are two distinct and independent strategies, each with its own theoretical and historical facets, which share some common mechanisms at both functional and neurobiological levels [111–113]. Both strategies employ attentional skills [112, 113] and use an attentional focus to develop the ability to be mindful (in mindfulness) or becoming immersed in suggestion-related experiences (in hypnosis) [111]. Based on these and other similarities (please refer to Otani et al. [112] for a comprehensive examination of common mechanisms between these two approaches), some authors have suggested to combine them, in order to use hypnosis to enhance the effects of mindfulness [114–116] and coining the term “mindful hypnotherapy” [117].

Other authors have instead underlined that mindfulness and hypnosis could not be considered as overlapping constructs [111, 112, 118]. As specified by Grover et al. [118], the purpose of hypnosis is to experience changes in consciousness and behavior as a result of suggestive induction. In contrast, the purpose of mindfulness is to notice what is happening in the unfolding experience of sensations, emotions, and thoughts, moment by moment, without the intention to promote an imminent change. Suggestion could be considered as a common mechanism of both strategies, but with different targets: mindfulness promotes a change in the relationship with the experience, while hypnosis fosters a change in the experience itself [118]. Recently, Grover et al. [118] have shown that higher levels of hypnotizability are associated with a reduction of mindfulness facets, in particular the ability to observe, non-react, and non-judge, suggesting that the two strategies have distinct effects and could therefore be offered according to the predominant characteristics of the subjects.

In conclusion, although the two approaches have a long tradition of use, it is only in recent years that clinicians and researchers have started to investigate their common aspects. Therefore, further research is needed to assess whether and how these two strategies can be combined to enhance overall clinical effectiveness.

## Conclusions

Fighting the obesity epidemic is challenging due to the ineffectiveness of diet and exercise alone, so finding effective new strategies is mandatory. Due to the relevant psychological involvement in the pathogenesis of obesity, different psychological strategies applied alone or in combination seem to offer a greater chance of success. Hypnosis and mindfulness are ancient strategies that in recent years have gained renewed interest, due to the spread of the offer, the variety of therapeutic applications, and the flexibility of use, which also includes self-administration. Both hypnosis and mindfulness provided additional benefit in the treatment of obesity when applied in a weight management program with or without other psychological interventions; however, owing to the heterogeneity of hypnosis and mindfulness strategies and the short-term duration of most studies, the relative evidence is at present scarce. Additional research is needed to determine whether these strategies are effective in the long term and whether they can be routinely introduced into the clinical practice.

## Abbreviations

ACT: Acceptance and commitment therapy
CBT: Cognitive-behavioral therapy
CFT: Compassion-focused therapy
DBT: Dialectical behavior therapy
MARS: Mobile App Rating Scale
MBIs: Mindfulness-Based Interventions
MBCT: Mindfulness-Based Cognitive Therapy
MB-EAT: Mindfulness-Based Eating Awareness Training
MBSR: Mindfulness-Based Stress Reduction Program
RCT: Randomized controlled trial
